# NOTIFICATION: The Role of Pathologists in Recognition of Morphologic and Biologic Features of Genetically Mutated Breast Cancer

**DOI:** 10.1155/tbj/9823045

**Published:** 2025-01-13

**Authors:** The Breast Journal

**NOTIFICATION:** S. Masood, “The Role of Pathologists in Recognition of Morphologic and Biologic Features of Genetically Mutated Breast Cancer” *The Breast Journal* 26, no. 8 (2020): 1583–1588, https://doi.org/10.1111/tbj.14012.

This notification is for the above article, published online on 26 August 2020 in Wiley Online Library (wileyonlinelibrary.com), and has been issued by agreement between the journal Editor-in-Chief, Dr. Guan-Jun Yang, and John Wiley & Sons Ltd. Following an investigation, the parties learned that an independent peer review process was not conducted for this article prior to publication. The current Editor-in-Chief of the journal, Dr. Guan-Jun Yang, has subsequently reviewed the content of the article and determined that it is suitable to remain published, noting that it does not represent original research, but primarily an editorial contribution from the previous Editor-in-Chief, Dr. Shahla Masood. Therefore, the parties have decided to issue this notification to inform and alert readers.

